# Bioinformatics and systems biology approaches to identify molecular targeting mechanism influenced by COVID-19 on heart failure

**DOI:** 10.3389/fimmu.2022.1052850

**Published:** 2022-11-07

**Authors:** Kezhen Yang, Jipeng Liu, Yu Gong, Yinyin Li, Qingguo Liu

**Affiliations:** School of Acupuncture-Moxibustion and Tuina, Beijing University of Chinese Medicine, Beijing, China

**Keywords:** COVID-19, heart failure, bioinformatics analysis, systems biology approaches, immunology

## Abstract

Coronavirus disease 2019 (COVID-19) caused by the severe acute respiratory syndrome coronavirus 2 (SARS-CoV-2) has emerged as a contemporary hazard to people. It has been known that COVID-19 can both induce heart failure (HF) and raise the risk of patient mortality. However, the mechanism underlying the association between COVID-19 and HF remains unclear. The common molecular pathways between COVID-19 and HF were identified using bioinformatic and systems biology techniques. Transcriptome analysis was performed to identify differentially expressed genes (DEGs). To identify gene ontology terms and Kyoto Encyclopedia of Genes and Genomes pathways, common DEGs were used for enrichment analysis. The results showed that COVID-19 and HF have several common immune mechanisms, including differentiation of T helper (Th) 1, Th 2, Th 17 cells; activation of lymphocytes; and binding of major histocompatibility complex class I and II protein complexes. Furthermore, a protein-protein interaction network was constructed to identify hub genes, and immune cell infiltration analysis was performed. Six hub genes (*FCGR3A, CD69, IFNG, CCR7, CCL5*, and *CCL4*) were closely associated with COVID-19 and HF. These targets were associated with immune cells (central memory CD8 T cells, T follicular helper cells, regulatory T cells, myeloid-derived suppressor cells, plasmacytoid dendritic cells, macrophages, eosinophils, and neutrophils). Additionally, transcription factors, microRNAs, drugs, and chemicals that are closely associated with COVID-19 and HF were identified through the interaction network.

## Introduction

Coronavirus disease 2019 (COVID-19), caused by severe acute respiratory syndrome coronavirus 2 (SARS-CoV-2), remains prevalent worldwide. All continents have reported cases of COVID-19 since the pandemic began. As of August 30, 2022, there have been approximately 600 million confirmed cases and more than 6.46 million deaths worldwide (https://covid19.who.int/). It has been found that patients with pre-existing cardiovascular disease who are infected with SARS-CoV-2 may be at increased risk of severe illness and death (1–3). Although COVID-19 primarily affects the respiratory system, severe cardiovascular damage has also been found, and there is a higher risk of death in patients with underlying cardiovascular disease (4). Cardiovascular complications, including heart failure (HF), arrhythmia, myocardial injury, and acute coronary syndrome, have been reported in approximately 14.1% of patients during hospitalization (5), with an overall HF incidence up to 14% (6). COVID-19 may induce HF; however, the exact mechanism remains unknown.

In this study, we investigated the association between COVID-19 and HF. COVID-19 causes changes in various immune cells (7). Moreover, COVID-19 is associated with a high inflammatory state and cytokine storm characterized by high levels of pro-inflammatory cytokines such as IL-1β, IL-6, and monocyte chemoattractant protein-1 (MCP-1) (8). This systemic release of cytokines may lead to T cell dysregulation and subsequent systemic inflammation, resulting in cardiac injury and multi-organ damage. There is evidence that T cell subsets have the potential to become biomarkers for HF which can be reduced by inhibiting the activation of some T cell subsets (9). During HF, immune cells can infiltrate myocardial tissues and release pro-inflammatory cytokines (10). Elevated serum IL-6 levels predict mortality in approximately 50% of HF patients (11), and high tumor necrosis factor (TNF)-α levels in patients with severe COVID-19 may exacerbate the development of HF (12). Although there is no direct experimental evidence explaining the molecular mechanisms by which COVID-19 induces HF, there may be some similarities in the immune mechanisms between COVID-19 and HF. Neutrophils, macrophages, and CD4+T cells in patients with COVID-19 can infiltrate myocardial tissue, causing pathological cardiac remodeling and fibrosis, leading to the development of HF and increased mortality (13). Several autopsies have reported macrophage and CD4+T cell infiltration in the myocardial tissue of patients with COVID-19 (14). SARS-CoV-2 has also been found in macrophages from the hearts of patients with COVID-19, suggesting that the virus can directly infect cardiomyocytes (15). This evidence suggests that the immune cells are altered during COVID-19 and HF.

**Figure 1:**
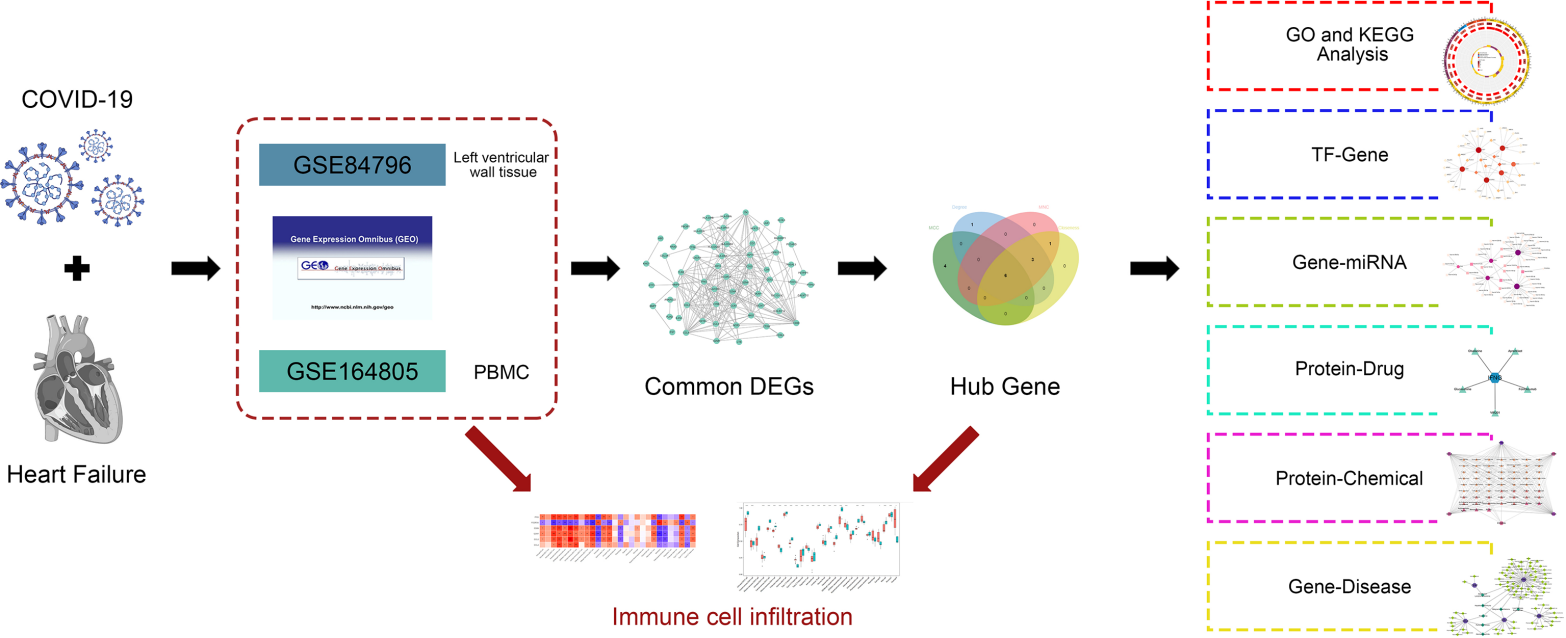
Schematic diagram of the workflow in this study.

In this study, we obtained data on patients with COVID-19 and HF from the gene expression omnibus (GEO) database. Using common differentially expressed genes (DEGs) between COVID-19 and HF, a protein–protein interaction (PPI) network was constructed and hub genes were identified. Gene ontology (GO) and Kyoto Encyclopedia of Genes and Genomes (KEGG) pathway analyses identified important terms and pathways associated with COVID-19 and HF. Notably, combined with immune cell infiltration analysis, we explored the correlation between immune cells involved in COVID-19 and hub genes. In addition, the interaction network of transcription factors (TFs), microRNAs (miRNAs), drugs, and chemicals was constructed using hub genes. The exploration of immune infiltration and multiple interaction networks of hub genes between COVID-19 and HF provides new insights into potential mechanisms and therapeutic targets. The analysis process of this study is shown in **Figure 1**.

## Methods

### Acquisition of disease gene datasets

To determine the shared genes between COVID-19 and HF, we obtained the COVID-19 and HF disease gene dataset from the National Center for Biotechnology Information (NCBI) GEO database (https://www.ncbi.nlm.nih.gov/geo/). The dataset associated with COVID-19 was GSE164805 (16), platform number GPL26963. It was developed using peripheral blood mononuclear cells from 10 patients with COVID-19 and five healthy controls. The dataset related to HF was GSE84796 (17), platform number GPL14550. It was developed using left ventricular wall tissue from the hearts of 10 patients with HF and seven healthy controls at the time of heart transplantation. The basic patient characteristics of the dataset are shown in **Table 1**. Both disease datasets were analyzed for genetic data from all samples using a microarray of genome-wide transcriptomes.

**Table 1:** Demographic characteristics of study population.

	GSE164805		GSE84796	
	COVID-19n=10	Healthn=5	Heart Failuren=10	Healthn=7
Age, years	54.7 ± 7.57	59.8 ± 6.94	50.2 ± 9.95	27.6 ± 11.06
Male	9	4	5	7
Female	1	1	5	0

COVID-19: coronavirus disease 2019.

### Identification of DEGs

Different conditions cause statistically significant differences in transcript levels of a gene; when this occurs, the gene is described as a DEG (18). The two datasets were screened separately for DEGs using the Limma package of the R software (version 4.2.0), resulting in the identification of genes most closely associated with disease development. The screening criteria were P-value<0.01 and |logFC|>2 for the COVID-19 dataset, and P-value<0.05, and |logFC|>1.5 for the HF dataset. Different screening criteria were based on the number of DEGs. The DEGs common to both COVID-19 and HF were common DEGs and more closely related to the development of both genes. A Venn diagram was used to demonstrate the identification process of common DEGs.

### GO and KEGG pathway enrichment analysis

To explore the common DEGs involved in the development of COVID-19 and HF in terms of common terms and pathways, GO and KEGG enrichment analyses were performed using Metascape (https://metascape.org/). The only species selected was “Homo sapiens,” and the screening criteria were Min Overlap=3, P-value Cutoff=0.01, and Min Enrichment=1.5. The results of the enrichment analysis were visualized using the “ggplot2” program of the R software. The top 25 pathways in the KEGG enrichment analysis are shown according to the P-values. GO enrichment analysis contained three sections, namely, biological process (BP), cell component (CC), and molecular function (MF), showing the top 10 terms with the smallest P-value in each section.

### Protein-protein interaction network analysis

After a cellular protein is synthesized, it forms a PPI structure similar to a subordinate relationship to function, which indicates the protein mechanism. By constructing PPI networks and evaluating their functions, key nodes and important targets for the operation of cellular mechanisms can be better explained (19–21). Common DEGs for COVID-19 and HF were used to construct PPI networks using the STRING database (https://cn.string-db.org/) and describe the functional and physical interactions. The species restriction was “Homo sapiens” and PPI information was extracted using an interaction score of 0.4. PPI network visualization was performed using Cytoscape 3.9.1 (http://cytoscape.org/.ver.3.9.1). Cytoscape is an open-source network visualization platform for any molecular composition and interaction system that allows for the rapid development of computational analysis capabilities (22).

### Identification of hub genes

A PPI network consists of nodes and edges, where nodes represent molecular components and edges connect different nodes, indicating interactions between neighboring nodes. The nodes that are more associated with other nodes are called hub genes in the PPI network. To explore the hub genes in common DEGs that were most closely associated with COVID-19 and HF, CytoHubba (http://apps.cytoscape.org/apps/cytohubba), a Cytoscape plug-in, was used. CytoHubba can extract central elements based on network features, uses multiple computational methods, and generally combines multiple analysis methods. We used the MCC, degree, MNC and closeness methods in CytoHubba to identify the top 10 rated genes from the PPI network, and the results of the four methods were used to determine the intersection and identify the hub genes.

### Assessment of immune cell infiltration and its correlation with common DEGs

Immune cells adopt specific programs of expression based on the tissue environment in which they infiltrate (23). Understanding the infiltration status of different immune cells in tissues can better explain the role played by immune cells and their role in pathogenesis of the disease. The ssGSEA algorithm in the GSVA package of the R software was used to quantify 28 immune cells in the COVID-19 dataset GSE164805 and obtain 28 immune cell expression matrices. To analyze how COVID-19 induces HF, we used hub genes closely related to COVID-19 and HF, combined with immune cell infiltration analysis, to calculate Spearman correlations. Immune infiltration was visualized using the ggplot2 software package.

### Identification of TFs and miRNAs

TFs recognize specific DNA sequences to control transcription as well as play an important role in directing gene expression, which determines how cells function and respond to their environment, making TFs one of the key cellular components that control gene expression (24, 25). miRNAs can be involved in the regulation of protein expression by binding to one or more sites on the mRNA transcription sequence and inhibiting translation, either directly by acting on the target gene or indirectly by first regulating the TF and subsequently controlling gene expression (26, 27). NetworkAnalyst is an online analysis platform that integrates publicly available and unrestricted access to multiple databases for network analysis of genes and multiple molecular components (28). Using the NetworkAnalyst platform, we found TFs associated with hub genes from the JASPAR database and built a TF–gene interaction network, as well as miRNA data from the TarBase (v8.0) database and built a Gene–miRNA interaction network. Visualization of the interaction networks was achieved using the Cytoscape software.

### Evaluation of protein interactions with drugs and chemicals

We searched for drugs and chemicals that were closely related to COVID-19 and HF by predicting the interaction network between proteins and drugs or chemicals. The protein–chemical network was implemented using the NetworkAnalyst platform to obtain protein–chemical correspondence from the comparative toxicogenomics database (CTD). In addition, drugs associated with proteins were identified from the DrugBank database through the NetworkAnalyst platform. The results were visualized using Cytoscape to identify the drugs and chemicals corresponding to multiple proteins.

### Analysis of gene-disease interaction networks

The exploration of genetic information regarding human diseases is a key point in precision medicine and drug discovery (29). DisGeNET integrates and standardizes data from multiple databases of genes and disease variants (30). Using the NetworkAnalyst platform, we discovered the relationship between genes and diseases, revealing the diseases associated with COVID-19 and HF.

## Results

### Identification of common DEGs in COVID-19 and HF

**Figure 2:**
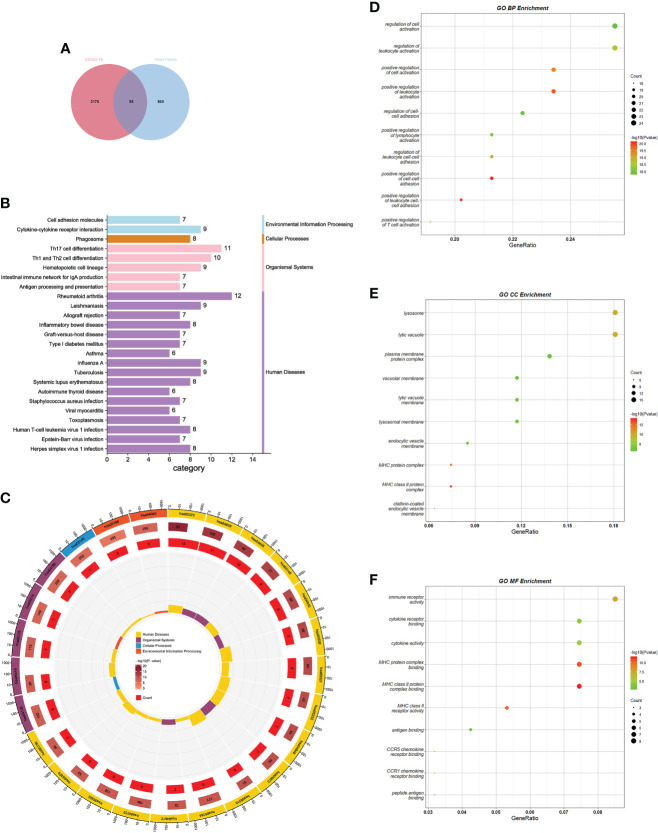
GO and KEGG pathway enrichment analysis of common DEGs. **(A)** Screening process of common DEGs. **(B)** KEGG enrichment analysis bar graph. Horizontal coordinates indicate the number of genes annotated to this pathway, and different colors indicate different pathway classifications. **(C)** KEGG enrichment analysis circle diagram. The first circle indicates the first 25 pathways, and the number of genes corresponds to the outer circle. The second circle indicates the number of genes and P-values in the genomic background. The third circle indicates the number of genes annotated to this pathway. The fourth circle indicates the enrichment factor for each KEGG term. **(D–F)** GO enrichment analysis bubble chart. The size of the dots corresponds to the number of genes annotated to this term, and the color of the dots corresponds to the magnitude of the P-value. GO, gene ontology; DEGs, differentially expressed genes; KEGG, Kyoto encyclopedia of genes and genomes.

To test the interrelationship and significance between COVID-19 and HF, we analyzed microarray datasets from the GEO database. Screening of DEGs was performed using the Limma package in R language with adjusted P-values by Benjamin-Hochberg. The screening criteria used for the COVID-19 dataset GSE164805 were p-value<0.01 and |log FC|>2. A total of 3270 DEGs were identified, including 1833 upregulated and 1437 downregulated genes. The screening criteria used for the HF dataset GSE84796 were p-value<0.05, |log FC|>1.5, and 964 DEGs were identified, including 753 upregulated and 211 downregulated genes. Cross-comparison analysis using Venn plots was performed to determine the intersection of DEGs between the two diseases, and 95 common DEGs were identified (**Figure 2A**). The common DEG set was used for subsequent analyses.

### GO and KEGG pathway enrichment analysis

GO and KEGG analyses were performed using Metascape to determine the biological significance of common DEGs in COVID-19 and HF. KEGG is a database that integrates genomic, chemical, and phylogenetic information. KEGG enrichment analysis, a modeling technique for understanding the high-level functions and utilities of biological systems by analyzing molecular-level information (generally large-scale molecular datasets), demonstrates the interactions between various diseases through underlying molecular or biological processes (31). We collected the 25 pathways with the smallest p-values and presented them using bar and circle plots (**Figures 2B, C**). Among the enriched results, most pathways were found to be related to immune responses, including diseases dominated by immune responses, diseases caused by autoimmune abnormalities, antigenic changes during immune responses, and immune cell changes.

GO is a freely accessible and unrestricted repository that mainly considers the functions of genes and provides computationally available resources. GO consists of three interrelated structured vocabulary categories: BP, CC, and MF. By annotating the structured vocabulary terms of each category; it is possible to understand how these terms fit together to form a coherent biological context (32). Our analysis summarized the 10 terms with the smallest p-values in each category for BP, CC, and MF. The enrichment results of BP mainly revealed activation and regulation of leukocytes, T cells, and lymphocytes. The enrichment results for CC mainly involved major histocompatibility complex (MHC)-like protein complexes. The enrichment results for MF highlighted the binding of the MHC-like protein complexes, antigen binding, and immune factor activity. All the results were visualized using bubble plots (**Figures 2 D–F**). The results of the enrichment analysis suggested a close association between common DEGs and immune responses in patients with COVID-19 and HF.

### Identification of hub genes

**Figure 3:**
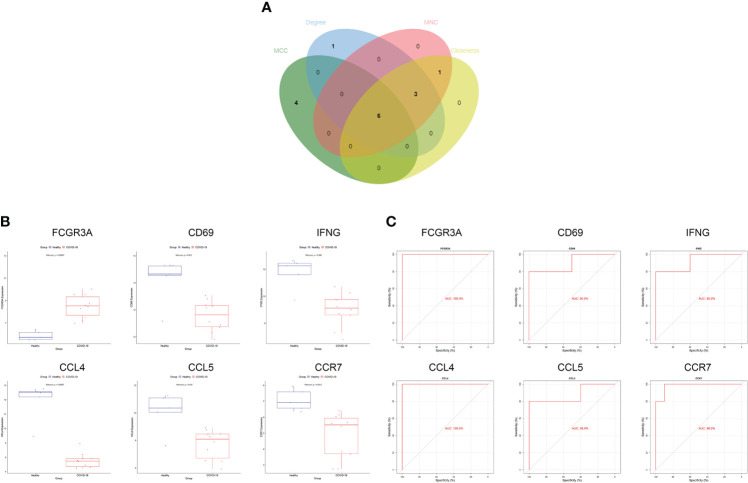
Screening and validation of hub genes. **(A)** Screening process of hub genes. **(B)** Expression of hub genes in the COVID-19 dataset; blue and red indicate the healthy and COVID-19 groups, respectively. Expression of FCGR3A was significantly higher and that of CD69, IFNG, CCR7, CCL5, CCL4 was significantly lower in COVID-19 compared to expression levels in the healthy group. **(C)** AUC values of hub genes in the COVID-19 dataset. ROC curves and AUC statistics were used to assess the ability to distinguish COVID-19 from healthy controls with excellent sensitivity and specificity. COVID-19: coronavirus disease 2019. AUC, area under the curve; ROC, receiver operating characteristic.

We generated PPI networks of common DEGs using STRING and imported the results into Cytoscape for further analysis to predict gene interactions. The CytoHubba plugin can rank nodes in a network based on network characteristics, and 11 topological analysis methods are commonly used to analyze hub genes in networks (33). We used four topological methods for hub gene screening: MCC, degree, MNC, and closeness. The top 10 genes were obtained for each algorithm score, and the genes shared by the four algorithms were Fc gamma receptor IIIa (*FCGR3A*), cluster of differentiation 69 (*CD69*), interferon-γ (*IFN-γ*; *IFNG*), C-C chemokine receptor type 7 (*CCR7*), chemokine ligand 5 (*CCL5*), and CCL4 (**Figure 3A**). The expression levels of the hub genes in the COVID-19 dataset are shown using box-line plots (**Figure 3B**). *CD69, IFNG, CCR7, CCL5*, and *CCL4* were found to be downregulated in the disease group, and *FCGR3A* was upregulated in the disease group, with statistically significant differences in the expression levels of each hub gene compared to the normal group (p<0.05). In the receiver operating characteristic (ROC) curve analysis, the area under the curve (AUC) values of the Hub genes were compared to assess their sensitivity and specificity for COVID-19. The AUC values of all six hub genes were >0.85, indicating that these genes have high diagnostic value for COVID-19 (**Figure 3C**). These hub genes are closely associated with both COVID-19 and HF and are involved in the development of both diseases. Validation of the hub genes was not performed because no suitable disease gene dataset was found.

### Immune cell infiltration and its correlation with hub genes

**Figure 4:**
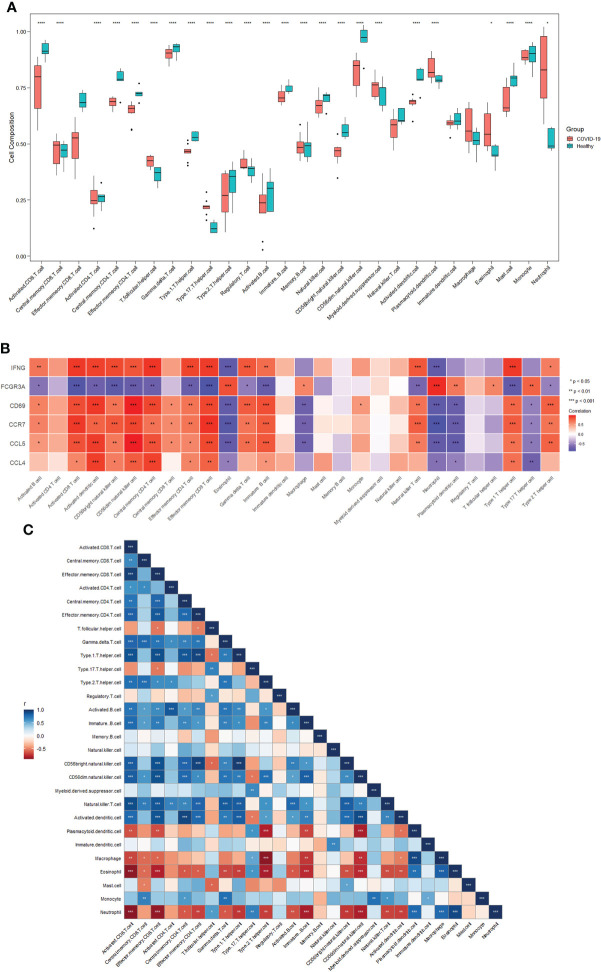
Immune cell infiltration analysis. **(A)** Distribution of 28 immune cells in the healthy and COVID-19 groups. **(B)** Correlations between six hub genes and different immune cells. Red color indicates positive correlation; blue color indicates negative correlation. **(C)** Correlation between 28 immune cells. Red indicates positive correlations; blue indicates negative correlations. *p<0.05, **p<0.01, ***p<0.001, ****p<0.0001. COVID-19: coronavirus disease 2019.

To further investigate differences in immune cell infiltration between patients with COVID-19 and healthy controls, the ssGSEA algorithm was used to assess the relationship between the two groups of samples. **Figure 4A** shows the distribution of 28 immune cells in the two groups of samples, and the results show that central memory CD8 T cells, T follicular helper (Tfh) cells, regulatory T cells (Tregs), myeloid-derived suppressor cells (MDSCs), plasmacytoid dendritic cells (pDCs), macrophages, eosinophils, and neutrophils infiltrated significantly more the tissues of patients with COVID-19 than normal tissues (p<0.05), suggesting that these immune cells are essential for the development of COVID-19. Combining immune cell infiltration analysis with the hub gene reveals which immune cells a hub gene is associated with. In connection with the COVID-19 gene dataset, these immune cells may be components of the mechanism through which COVID-19 induces HF.

**Figure 4B** shows the correlation between the hub genes and immune cells. Central memory CD8+T cells were positively correlated with *CD69* (cor=0.577, p=0.024), *CCR7* (cor=0.581, p=0.023), and CCL5 (cor=0.585, p=0.022). Tfh cells were positively correlated with *FCGR3A* (cor=0.602, p=0.017). pDCs were positively correlated with *FCGR3A* (cor=0.660, p=0.007) and negatively correlated with *CD69* (cor=-0.745, p=0.001), *CCR7* (cor=-0.766, p<0.001), *CCL5* (cor=-0.785, p<0.001), and *CCL4* (cor=-0.535, p=0.040). Macrophages were positively correlated with *FCGR3A* (cor=0.598, p=0.019) and negatively correlated with *CD69* (cor=-0.717, p=0.003), *CCR7* (cor=-0.687, p<0.005), and *CCL5* (cor=-0.713, p<0.003). Eosinophils were positively correlated with *FCGR3A* (cor=0.829, p<0.001) and negatively correlated with *IFNG* (cor=-0.801, p<0.001), *CD69* (cor=-0.782, p<0.001), *CCR7* (cor=-0.853, p<0.001), *CCL5* (cor=- 0.776, p<0.001), and *CCL4* (cor=-0.544, p=0.036). Neutrophils were positively correlated with *FCGR3A* (cor=0.941, p<0.001) and negatively correlated with *IFNG* (cor=-0.789, p<0.001), *CD69* (cor=-0.796, p<0.001), *CCR7* (cor=-0.895, p<0.001), *CCL5* (cor=- 0.781, p<0.001), and *CCL4* (cor=-0.605, p=0.017). No significant correlation was observed between Tregs or MDSCs and hub genes.

**Figure 4C** shows the correlation between the 28 immune cells. Central memory CD8+T cells were positively correlated with macrophages (p<0.05) and eosinophils (p<0.05). Tfh cells were negatively correlated with Tregs (p<0.05) and neutrophils (p<0.05). pDCs were negatively correlated with macrophages (p<0.001), eosinophils (p<0.01), and neutrophil (p<0.001). Macrophages were negatively correlated with eosinophils (p<0.001) and neutrophils (p<0.001). Eosinophils levels were negatively correlated with neutrophil counts (p<0.001). No significant correlation was observed between Tregs or MDSCs and other immune cells. These results demonstrate a clear correlation between hub genes and some immune cells. All changes in COVID-19 may be a potential mechanism for inducing HF.

### Gene regulatory network analysis of hub gene interactions with TFs and miRNAs

**Figure 5:**
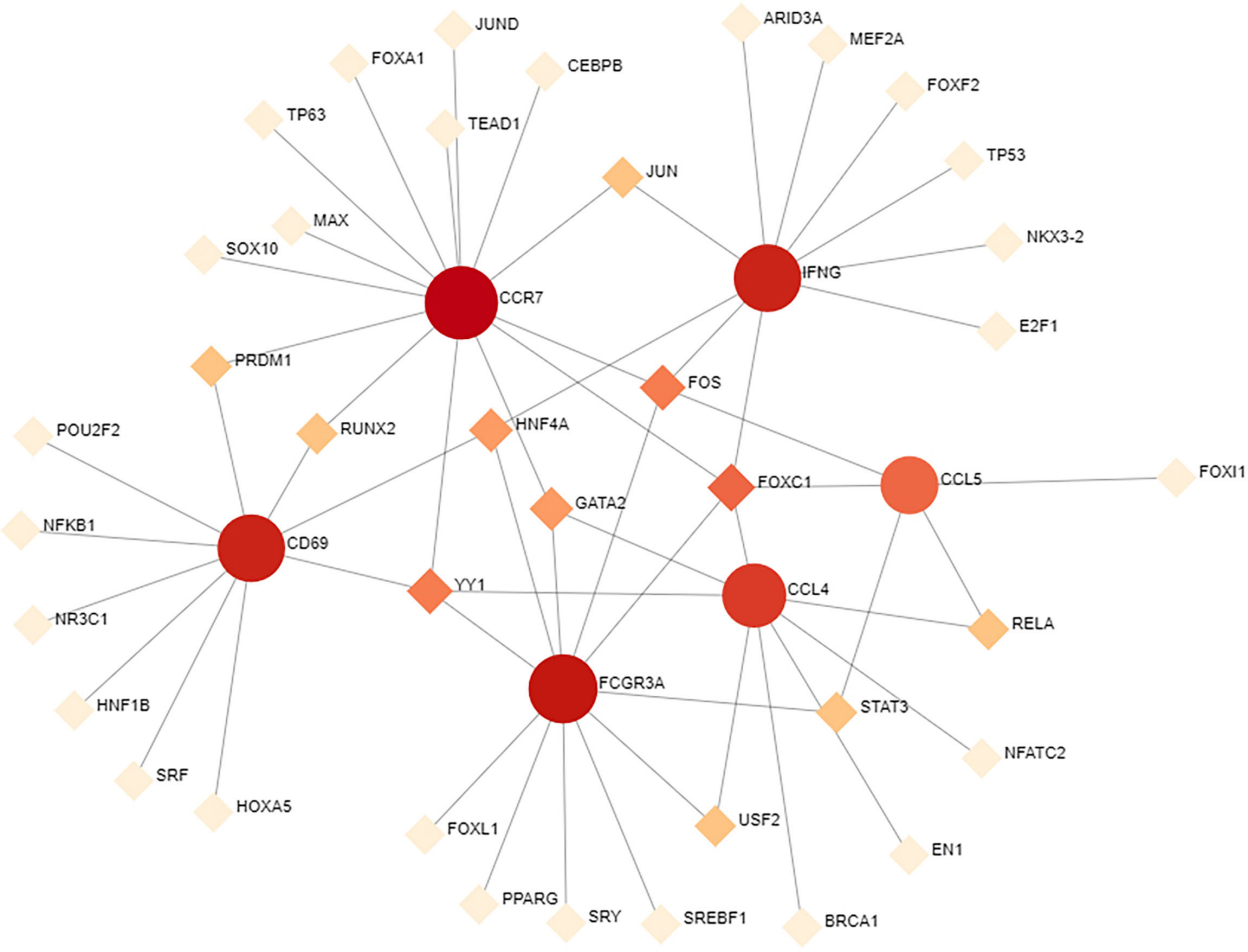
TF–gene interaction network analysis. Round dots indicate hub genes; square dots indicate TFs. Darker colors indicate association with a greater number of hub genes and a higher degree of association. TF, transcription factor.

**Figure 6:**
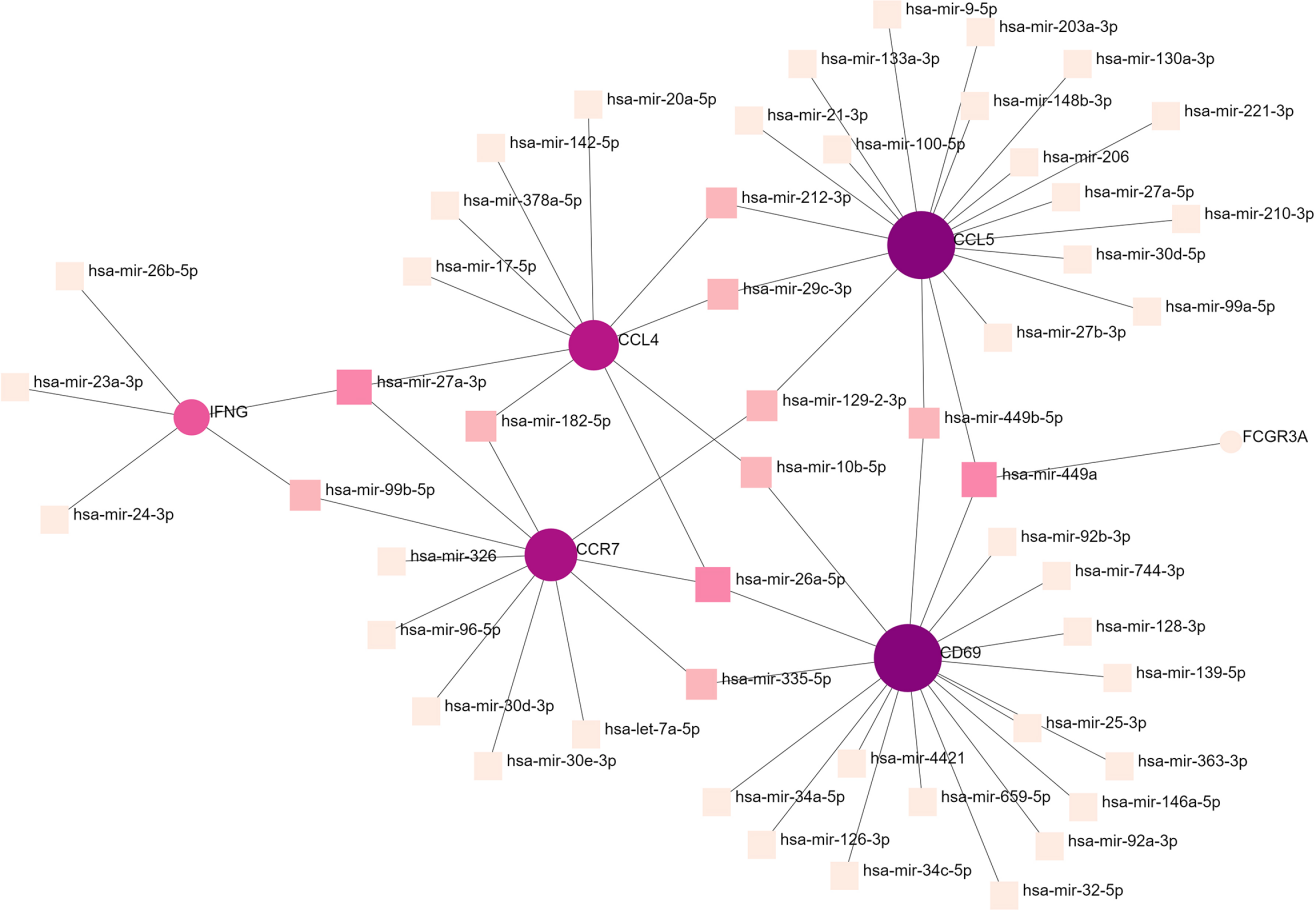
Gene–miRNA interaction network analysis. Round dots indicate hub genes; square dots indicate miRNAs. Darker colors indicate association with a greater number of hub genes and a higher degree of association. miRNA, microRNA.

To determine the substantial changes in hub genes at the transcriptional level, we analyzed the TFs and miRNAs closely associated with hub genes and constructed the TF–gene and gene-miRNA networks. **Figure 5** shows the TFs interacting with hub genes, with six hub genes corresponding to 38 TFs. Among them, the TFs associated with at least two hub genes were FOXC1, FOS, YY1, GATA2, HNF4A, STAT3, RELA, USF2, JUN, PRDM1, and RUNX2. This indicated that there were interactions between these transcription factors and multiple hub genes. **Figure 6** shows the miRNAs associated with hub genes, with six hub genes corresponding to 51 miRNAs. All hub genes except FCGR3A corresponded to multiple miRNAs. The miRNAs associated with multiple hub genes were hsa-mir-27a-3p, hsa-mir-26a-5p, hsa-mir-449a, hsa-mir-335-5p, hsa-mir-99b-5p, hsa-mir-182-5p, hsa-mir-129-2-3p, hsa -mir-10b-5p, hsa-mir-449b-5p, hsa-mir-212-3p, and hsa-mir-29c-3p. Based on TF–gene and gene–miRNA interaction network analyses, we noted that the regulatory features of 11 transcription factors and 11 post-transcriptional miRNAs were closely associated with more than one hub gene, which indicated strong interaction between them.

### Protein–drug and protein–chemical interaction networks

**Figure 7:**
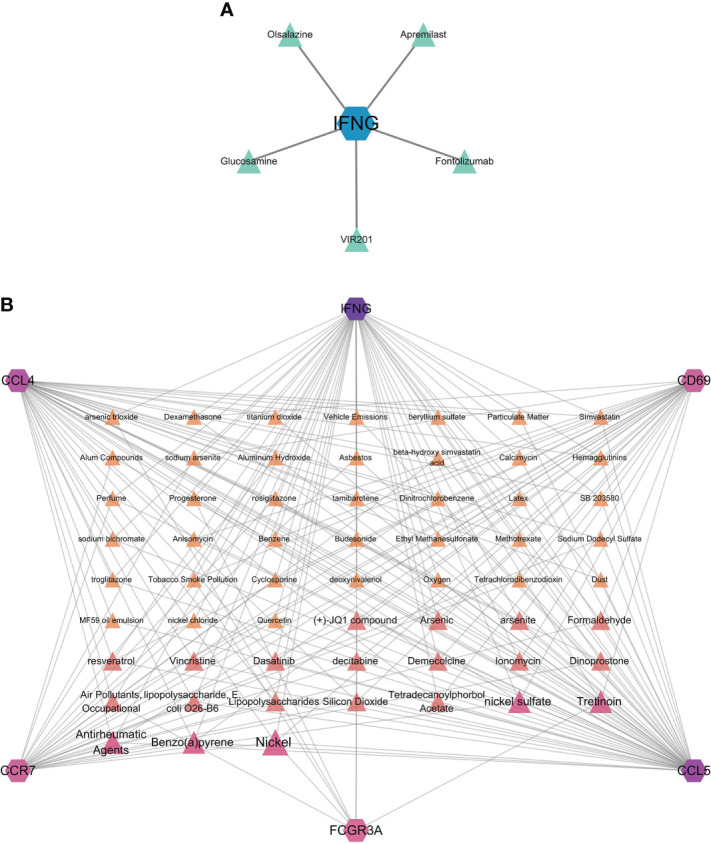
Protein–drug and protein–chemical interaction network analyses. **(A)** Drugs connected to the protein; blue dots indicate the protein, and green dots indicate the drug. **(B)** Chemicals linked to proteins. The diagram only shows chemicals linked to at least three proteins, with darker colors and larger font sizes indicating linkage to a greater number of proteins.

Hub genes identified in the interaction between COVID-19 and HF which were strongly associated with both diseases were used in this analysis. We identified protein**–**drug and protein**–**chemical interaction networks that may affect these hub genes. **Figure 7A** shows the protein**–**drug interactions, which were obtained from the DrugBank database. We did not other protein**–**drug relationships for any protein except IFNG. Drugs associated with IFNG may have potential as novel therapeutic agents. **Figure 7B** shows the protein**–**chemical interactions, and only chemicals associated with at least three hub genes are shown in the figure. The top five chemicals were nickel, nickel sulfate, benzo(a)pyrene, antirheumatic agents, and tretinoin, indicating that these chemicals are closely associated with COVID-19 and HF.

### Gene–disease interaction network

**Figure 8:**
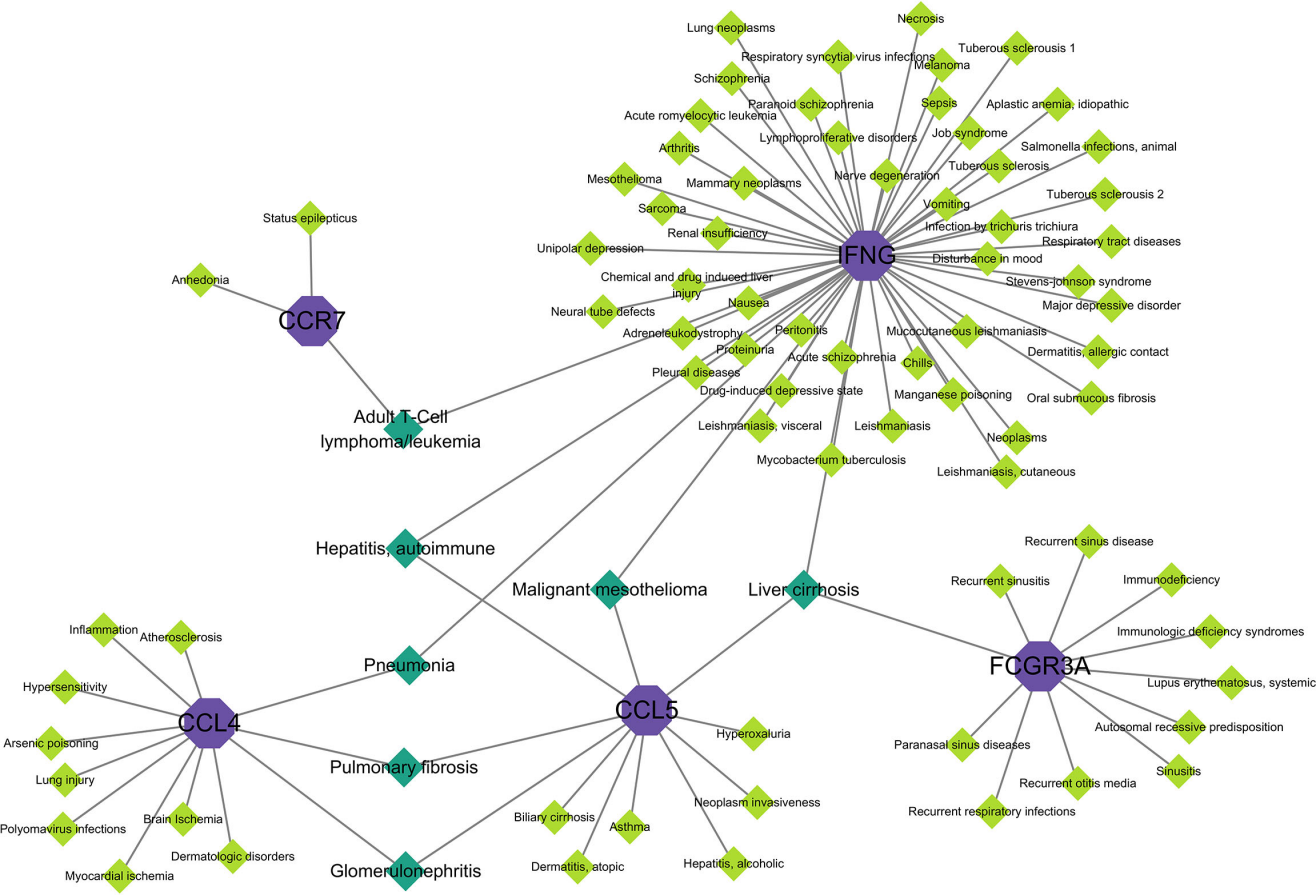
Gene–disease interaction network analysis. Purple dots in the graph indicate hub gene, green dots indicate diseases associated with hub genes, and dark green dots indicate diseases associated with multiple hub genes.

When different diseases are interrelated, one or more disease-associated genes or genes with similar sequences are generally linked to both diseases, and analysis of gene**–**disease associations can be used to elucidate the molecular mechanisms of disease (34). Explaining the association between genes and diseases can contribute to the design of therapeutic strategies for these diseases (35). **Figure 8** shows the associations between hub genes and diseases. In the interaction network, IFNG was associated with more diseases, whereas CD69 was not associated with any disease. The diseases associated with two hub genes included adult T cell lymphoma/leukemia, hepatitis, autoimmune disease, pneumonia, pulmonary fibrosis, glomerulonephritis, and malignant mesothelioma. Diseases associated with the three hub genes included liver cirrhosis. Most of these diseases are associated with the lungs, liver, and kidneys, in which the immune response has a crucial effect.

## Discussion

In this study, we used gene expression data (for COVID-19 and HF caused by Chagas disease) to identify relevant biomarkers and molecular targets. In the initial analysis, a percentage of patients from the COVID-19 dataset were identified as having hypertension and cardiovascular disease, which could be a potential risk for HF. The HF samples were obtained from patients with end-stage HF caused by severe Chagas chronic cardiomyopathy (severe dilated cardiomyopathy). We identified hub genes that may be associated with COVID-19-induced HF by comparing the common DEGs between the two datasets. Changes produced by the immune system in response to different foreign antigens may be similar. By linking a hub gene to COVID-19 through immune cell infiltration analysis, we identified immune pathways shared between COVID-19 and Chagas. This may indicate the mechanism of COVID-19-induced HF.

GO is a key resource for obtaining biological information, providing specific definitions of protein function, describing how genes function in biological systems, and describing terms in a computationally tractable manner. In BP, the results were mainly related to leukocytes, T cells, and lymphocytes, including the positive regulation of cell activation and cell–cell adhesion. The body’s adaptive immune system comprises three main cell types: B, CD4+T, and CD8+T cells. The immune response plays a direct role in HF and can act as a secondary effector, and activation of immune response mechanisms in the heart may cause HF (36). SARS-CoV-2 infection stimulates a series of immune responses in the body, with CD4+T cells responding more prominently to the virus than CD8+T cells (37). Compared to antibodies and CD8+T cells, specific CD4+T cells are more strongly associated with reduced COVID-19 severity (38). In the enrichment results for CC and MF, the terms MHC protein complex and MHC II protein complex were highly ranked, suggesting that they are more closely associated with COVID-19. During viral infection, T cells can recognize viral antigens delivered by MHC I protein complexes, which further promotes cytokine release and cytotoxicity through CD8+T cells (39). In some cases, MHC II protein complexes can also present viral peptides to CD4+T cells (40). SARS-CoV-2 suppresses antigen expression by downregulating expression of MHC I and II protein complexes, thereby inhibiting T cell-mediated immune responses (41). Among the top 25 pathways with the smallest p-values, many immune-related diseases were identified, including rheumatoid arthritis, inflammatory bowel disease, systemic lupus erythematosus, and autoimmune thyroid disease. Many immune-related pathways are presented in the results, including lymphocyte differentiation, antigen processing, and presentation. T helper (Th) 1, Th2, and Th17 cells play important roles in the immune response. Virus-specific CD4+T cells differentiate into Th1 cells, which produce IFNG and related cytokines, and thus, have antiviral activity, and Tfh cells, which assist B cells and are essential for neutralizing antibody responses and long-term humoral immunity (42). The results of GO and KEGG enrichment analyses suggest that these biological pathways related to the immune response may be involved in the progression of COVID-19 and HF disease.

COVID-19 is characterized by over-activation of the innate and adaptive immune systems, leading to a high inflammatory state and cytokine storm, which in turn causes organ damage (43). In our study, the results of immune cell infiltration analysis showed that the numbers of central memory CD8 T cells, Tfh cells, Tregs, MDSCs, pDCs, macrophages, eosinophils, and neutrophils were significantly higher in COVID-19 patients than in the healthy population (p<0.001). The actions of T cells in COVID-19 protection and pathogenesis are incomplete, unclear, and sometimes contradictory (44–47). CD8+ T cells play an important role in immune defense against foreign antigens. When foreign antigens are recognized, these cells secrete IFNG with an antiviral effect. In a study of T cells, a higher proportion of CD8+T cells than CD4+T cells was found in patients with mild COVID-19, suggesting a potential protective role for CD8+T cells in mild disease (48). Tfh cells are a specific subpopulation of CD4+T cells that play a key role in adaptive immunity and help B cells produce specific antibodies. Tregs control the immune response to self and foreign antigens and help prevent autoimmune diseases. MDSCs are defined as innate bone marrow-derived immune cells that inhibit effector T cell responses and are specialized immunosuppressive cells that control the function of other immune cells and prevent excessive inflammatory responses (49, 50). However, the increased number of MDSCs during COVID-19 may inhibit the action of T cells and promote viral survival (51). pDCs, which are found mainly in lymphoid organs, detect nucleic acids from pathogens and rapidly produce large amounts of type I IFN in response to viral detection (52). One study found that elevated pro-inflammatory cytokines promote macrophage activation, which may induce severe COVID-19 (53). Although eosinophils have potent antiviral activity become more abundant during COVID-19, there is no evidence that they have protective or worsening effects during viral infection (54). Neutrophils release neutrophil extracellular traps for viral inactivation (55) and promote cytokine production to limit viral replication (56). Lymphocyte counts decrease and neutrophil counts increase in patients with COVID-19 (57, 58). Furthermore, extensive neutrophil infiltration was found in the lung capillaries of patients with COVID-19 (59). However, severe COVID-19 reduces neutrophil, and eosinophil counts, suggesting that an abnormal, over-activated immune response may result in different outcomes (60). The pathogenic role of immune cell infiltration in exacerbating HF progression has been widely studied. An increased percentage of central memory CD8 T cells was found in patients with structural heart disease (61), and the Tfh cell population increased significantly in patients with HF due to ischemic cardiomyopathy (62). Pro-inflammatory and anti-angiogenic Tregs play an important pathogenic role in chronic ischemic HF by promoting immune activation (63). MDSCs can play a protective role in HF by exerting an antihypertensive effect on cardiomyocytes and producing anti-inflammatory effects through IL-6 and NO (64). Animal model studies have shown that dendritic cells activated and loaded with self-antigens are more likely to induce HF (65). HF stimulates the proliferation of macrophages in the heart (66), and different macrophage phenotypes are associated with HF regulation (67). In the early stages of HF, an increase in eosinophil count may have a protective effect on cardiomyocytes and is associated with better prognosis (68). Neutrophils play a crucial role in the pathogenesis and progression of HF, and neutrophil-mediated immune thrombotic dysregulation may be a key pathogenic mechanism leading to HF (69). These changes in the immune system may be associated with COVID-19-induced HF.

Through the PPI network of common DEGs, we identified six hub genes (*FCGR3A, CD69, IFNG, CCR7, CCL5*, and *CCL4*). Combining the immune cell infiltration data for COVID-19 with the hub genes, the correlation between hub genes and immune cells was further investigated to determine the immune response involved in the pathogenesis of HF, which may be a potential mechanism through which COVID-19 induces HF. Previous studies have shown a close association between hub genes and immune cells. FCGR3A promotes the production of pro-inflammatory cytokines and increases the activity of cytotoxic effector cells (70, 71). The SARS-CoV-2 immune complex can interact with FCGR3A and increase the production of pro-inflammatory cytokines (72, 73), and FCGR3A gene variants may exacerbate COVID-19 (74). In cardiovascular diseases, FCGR3A is a major checkpoint for the control of immune surveillance, and modulation of NK cell functions can help limit vascular injury in some patients (75). CD69 is a T-cell activation marker (76) which can regulate the adaptive immune response through different mechanisms, including prevention of Th17 differentiation and downregulation of pro-inflammatory cytokines (77–79). High expression of CD69 on CD4+ and CD8+T cells in COVID-19 leads to over-activation of NK cells and T cells, which may cause over-activation of the immune response (80). T cells have higher CD69 expression in patients with chronic HF, but this elevated expression may not be related to the etiology of HF (81). IFNG is a type II IFN mainly produced by NK cells. In the early stages of infection, macrophages bind to receptors on NK cells and secrete IL-12 leading to the production of IFNG. IFNG produced by NK cells, in turn, promotes the activation of phagocytes, resulting in an increase in IL-12 secretion by macrophages, forming a positive feedback regulation (82, 83). One study found that elevated levels of cytokines (IL-7, IL-15, and IL-2) in COVID-19 may promote IFNG production (84); however, IFNG-induced cell death may contribute to systemic inflammation and death in COVID-19 (85). Low IFNG levels have been reported in patients with severe COVID-19 (86). IFNG has the potential to regulate cardiac function, but it is unclear whether IFNG is deleterious or protective in the development of HF (87). CCL4 and CCL5 are chemokines. Chemokines interact with receptors on the cell surface to promote the recruitment of target cells, induce their movement to desired destinations and direct the chemotaxis of target cells (88). In a study on COVID-19, CCL4 was highly expressed in the bronchoalveolar lavage fluid of patients, and CCL5 expression was variable (89). In another study, CCL4 expression levels were lower in patients with COVID-19, particularly in severely ill patients (90). In patients with HF, high levels of CCL4 may lead to disease progression (91). Most inflammatory cells express CCL5 (92) and have been found to drive cell migration to cardiac tissue in patients with heart disease (93). CCL5 serum levels are significantly elevated in patients with HF (92), and by blocking CCL5, inflammation in the cardiac tissue can be targeted to reduce neutrophil and macrophage recruitment (94). During viral infection, antigen uptake by dendritic cells upregulates expression of CCR7, a G protein-coupled chemokine receptor, which drives antigen-presenting cells to lymphoid tissue (95). This process is critical for antigen uptake and presentation (96). In the early stages of COVID-19, a low proportion of naïve CCR7 CD4+T cells may be an independent early predictor of patient death (97). Chemokines are inflammatory signaling molecules that play an important role in the physiology of the heart and the stress response, and CCR7 has been found to be downregulated in HF patients (98). In the present study, the expression of some hub genes and the abundance of immune cells were different from those reported in previous studies. On the one hand, this may be because the abnormal immune response to COVID-19 is still unclear. On the other hand, this may coincide with the biological mechanisms that occur when COVID-19 induces HF, unlike when solely COVID-19 develops. These reasons may ultimately lead to the inconsistency of hub gene expression and immune cell abundance in the present study compared to previous studies. Therefore, the identified hub genes deserve further study, and they have the potential to become biomarkers for COVID-19 and HF that could help in clinical diagnosis and treatment or, if the pathogenesis of COVID-19 is further clarified, become new drug targets.

To further understand the role of hub genes in disease development, we constructed TF–gene and gene–miRNA networks. TFs can recognize specific DNA sequences to control transcriptional processes, forming a complex system that directs genomic expression (24). miRNAs are small endogenous non-coding RNAs. By identifying the target genes of miRNAs and further inferring their functions and regulatory mechanisms, it is possible to understand their normal biological functions and their key roles in disease pathophysiology (99). The TFs associated with all hub genes were FOXC1, FOS, YY1, GATA2, HNF4A, STAT3, RELA, USF2, JUN, PRDM1, and RUNX2. Based on previous studies, most of these 11 TF, especially FOXC1 and STAT3 are involved in the progression of COVID-19 and heart-related diseases (100). Eleven miRNAs were associated with all hub genes, some of which have been associated with liver cancer (101–103) (e.g., hsa-mir-27a-3p, hsa-mir-26a-5p, hsa-mir-182-5p, and hsa-mir-212-3p) and some have been closely related to lung cancer (104–107) (e.g., hsa-mir-335-5p, hsa mir-182-5p, hsa-mir-212-3p, and hsa-mir-29c-3p). In this study, most of the identified miRNAs were associated with cancer and may lead to different chronic cancers, such as colorectal cancer and prostate cancer. Only FCGR3A was found to be associated with a single miRNA. It has been reported that hsa-mir-449a participates in cancer development and may be a potential prognostic indicator (108). No correlation was found between FCGR3A and the other miRNAs, which warrants further investigation.

The relationship between hub genes, chemicals, and drugs was explored in our study. Olsalazine (109, 110) and fontolizumab (111) may be effective in treating active ulcerative colitis and may be beneficial for patients with Crohn’s colitis. Glucosamine is a nutritional supplement and a natural component of the cartilage that can be used to treat diseases, such as cerebral infarction (112) and arthritis (113), and exerts anti-inflammatory effects by inhibiting IL-1β. VIR201 is an experimental therapeutic vaccine used in clinical trials that improves immune function by stimulating IFNG expression. Apremilast is a phosphodiesterase 4 (PDE4) inhibitor (114) that alleviates diseases caused by inflammatory factors. In the present study, an association was found only between IFNG and certain drugs. These drugs exert anti-inflammatory or modulatory effects on the immune function, and IFNG may be a key element of the mechanism of action. In addition, we identified chemicals that were associated with all hub genes. In one study (115), a new nickel complex was found to exert an inhibitory effect on COVID-19 protease. Benzo(a)pyrene is a human carcinogen, and increased levels of benzo(a)pyrene in air can increase the risk of COVID-19 (116). The judicious use of antirheumatic agents can appropriately treat immune complications and reduce the morbidity and mortality associated with viral infections (117). Tretinoin, a retinoid used in the treatment of leukemia, reduces the replication of SARS-CoV-2 (118). Therefore, these chemicals deserve further investigation.

We identified diseases associated with hub genes based on the gene–disease interaction network. The results showed that COVID-19 combined with HF affected organs, including the brain, heart, lungs, liver, kidneys, blood, and skin, as well as psychiatric and immune-related diseases. Data suggest that SARS-CoV-2 infection may have severe pulmonary fibrotic consequences, with SARS-CoV-2 triggering a pro-fibrotic macrophage response and marked fibroproliferative acute respiratory distress syndrome (119). Malignant mesothelioma is a type of cancer caused by asbestos exposure. Malignant mesothelioma has been used to analyze the influence of psychological factors on COVID-19, and it was found that psychiatric disorders may be one of the influencing factors (120). Consistent with the results of the aforementioned studies, we found several psychiatric disorders associated with COVID-19 and HF, including schizophrenia, depressive disorders, and mood disturbances. COVID-19 also causes glomerular disease, which may be related to the immune dysregulation triggered by SARS-CoV-2 (121). Adult T cell lymphoma has been found in some patients with COVID-19, which is rare; cancer patients are immunocompromised, and therefore, are considered to be at high risk for severe disease associated with COVID-19. However, there is no direct evidence regarding immunosuppressive disease due to hematologic malignancies being a risk factor for severe COVID-19 (122).

## Limitation

This study has several limitations. The HF dataset we chose was caused by Chagas, which may have influenced the results of the study. The selection of different tissue samples for analysis may have caused differences. Although this study focused on the analysis of immune response in COVID-19 and HF, the identified hub genes could not be validated because of the absence of a suitable genetic dataset. The sample size was small, and thus, the results could be highly influenced by other variables and the presence of other conditions. The relationship between COVID-19 and HF is complicated and correlated; thus, further in-depth studies on the common pathogenetic mechanisms of both diseases are necessary to provide new insights for developing the appropriate therapeutic strategy.

## Conclusion

In conclusion, the present study identified common immune pathways between COVID-19 and Chagas-induced HF, which may be responsible for the induction of HF by COVID-19. The findings from bioinformatic and systems biology analyses emphasized that multiple immune cells were involved in the mechanism through which COVID-19 may induce HF. Six hub genes (*FCGR3A, CD69, IFNG, CCR7, CCL5*, and *CCL4*) were involved in regulating immune cells in COVID-19 and HF, which may contribute to the pathogenesis of HF. Furthermore, the results of enrichment analysis and different interaction networks revealed a common molecular mechanism between COVID-19 and HF. These results provide new insights into the clinical diagnosis and treatment of COVID-19 combined with HF.

## Data availability statement

Publicly available datasets were analyzed in this study. This data can be found here: https://www.ncbi.nlm.nih.gov/geo/query/acc.cgi?acc=GPL26963, https://www.ncbi.nlm.nih.gov/geo/query/acc.cgi?acc=GPL14550.

## Author contributions

KY: Conceptualization, Methodology, Investigation. JL: Resources, Writing – review and editing, Investigation. YG: Software, Validation. YL: Wu: Data curation, Writing – original draft. QL: Supervision, Project administration. All authors contributed to the article and approved the submitted version.

## Funding

This work was supported by the National Natural Science Foundation of China [grant number 81774413].

## Conflict of interest

The authors declare that the research was conducted in the absence of any commercial or financial relationships that could be construed as a potential conflict of interest.

## Publisher’s note

All claims expressed in this article are solely those of the authors and do not necessarily represent those of their affiliated organizations, or those of the publisher, the editors and the reviewers. Any product that may be evaluated in this article, or claim that may be made by its manufacturer, is not guaranteed or endorsed by the publisher.
